# Supplementary material for: An Improved Prediction Model for Ovarian Cancer Using Urinary Biomarkers and a Novel Validation Strategy
Source: Int J Mol Sci. 2019 Oct 5;20(19):4938. doi: 10.3390/ijms20194938 (PMC6801627; doi:10.3390/ijms20194938)
Supplement: Supplementary file 1 [file ijms-20-04938-s001.pdf]

Table S1. The estimated value of each single marker in the urine samples.

Markers	Cancer (N=158) [1]	Benign (N=125) [1]	t-score	P value
HE4 (pg/mL)	18.2 ± 2.58	15.3 ±1.57	11.40	1.26×10^{-24}
VCAM (pg/mL)	10.4 ± 2.64	7.8 ± 2.20	8.63	6.69×10^{-16}
TTR (ng/mL)	2.1 ± 2.17	0.1 ± 2.26	7.06	1.64×10^{-11}
Leptin (pg/mL)	5.1 ± 1.83	3.5 ± 1.53	6.70	2.18×10^{-10}
CRP (pg/mL)	7.7 ± 3.45	5.1 ± 3.09	6.11	4.35×10^{-9}
PDGF-AA (pg/mL)	4.1 ± 2.26	2.4 ± 2.46	5.95	9.18×10^{-9}
Cyfra21-1 (pg/mL)	11.9 ± 3.38	9.9 ± 2.65	5.32	2.30×10^{-7}
NCAM (pg/mL)	9.5 ± 1.41	8.6 ±1.47	5.07	7.43×10^{-7}
Prolactin (pg/mL)	4.6 ± 2.83	2.6 ± 2.35	4.78	4.79×10^{-6}
MPO (pg/mL)	11.0 ± 2.87	9.1 ± 3.84	4.27	2.87×10^{-5}
CEA (pg/mL)	10.4 ± 2.30	11.3 ± 1.92	-3.58	4.08×10^{-4}
Creatinine (nmole)	7.6 ± 1.75	8.3 ± 1.57	-3.53	4.85×10^{-4}
Mesomark (nM)	2.3 ± 2.56	0.8 ± 2.31	3.40	9.95×10^{-4}
CA19-9 (u/mL)	4.5 ± 2.25	3.6 ± 2.43	3.07	2.42×10^{-3}
PAI-1 (pg/mL)	2.8 ± 3.43	4.1 ± 4.62	-2.54	1.18×10^{-2}
ApoAI (ng/mL)	7.4 ± 2.38	6.7 ± 1.94	2.37	1.86×10^{-2}
MIF (pg/mL)	7.6 ± 2.63	6.9 ± 2.14	2.23	2.68×10^{-2}
IL-6 (pg/mL)	1.3 ± 2.39	0.7 ± 2.20	1.24	0.222
ApoCIII (ng/mL)	10.0 ± 3.03	9.6 ± 2.84	1.16	0.246
IL-8 (pg/mL)	2.2 ± 2.75	1.9 ± 3.15	0.78	0.434
CA15-3 (pg/mL)	5.1 ± 1.82	5.0 ± 1.71	0.69	0.494
OPN (pg/mL)	17.2 ± 2.43	17.2 ± 2.52	0.24	0.808
CA-125 (u/mL)	1.4 ± 2.26	1.4 ± 2.10	0.08	0.939

[1] mean ± standard deviation
